# Peripheral nerve injury with Nexplanon removal: case report and review of the literature

**DOI:** 10.1186/s40834-018-0070-0

**Published:** 2018-10-22

**Authors:** Rachel Lefebvre, Marianne Hom, Hyuma Leland, Milan Stevanovic

**Affiliations:** 1 Department of Orthopaedic Surgery, Division of Hand Surgery, University of Southern California, 1200 N State Street A7-CT, Los Angeles, CA 90033 USA; 2 Department of Obstetrics and Gynecology, University of Southern California, 2020 Zonal Ave., IRD Room 512, Los Angeles, CA 90033 USA; 3 Department of Plastic and Reconstructive Surgery, University of Southern California, 1510 San Pablo St., Suite 415, Los Angeles, CA 90033 USA

**Keywords:** Contraception complications, Iatrogenic peripheral nerve injury, Implant migration, Neuroma

## Abstract

**Background:**

Implantable devices offer convenient, long-acting, and reversible contraception. Injury to the peripheral nerves and blood vessels have been reported as rare complications of implantation and extraction.

**Case presentation:**

We present a case of ulnar nerve injury in a 21-year-old woman from attempted in-office removal of a deeply implanted Nexplanon® device. The injury resulted in an ulnar nerve palsy requiring surgical exploration, neuroma excision, and sural nerve cable grafting.

**Conclusions:**

In-office attempts to remove contraceptive implants that are deep or have migrated can cause iatrogenic nerve injury. Devices that are non-palpable, deep, or migrated should be imaged before formal surgical exploration and removal. Any patient with neurologic symptoms after placement or after attempted removal requires prompt diagnosis and referral to a peripheral nerve surgeon.

## Background

Subdermal contraceptive implants, such as the Nexplanon® (Merck, Kenilworth, NJ), are a popular form of long-acting, reversible contraception. Insertion and removal procedures are designed to be safely performed in the outpatient setting with local anesthetic. However, serious complications, including neurovascular injury, can occur [1–6]. These complications were more commonly reported with older devices, especially if they were placed deeply or if they migrated proximally. Multiple case reports have described implant-related injuries to the median [2, 3], ulnar [4, 5], and medial antebrachial cutaneous nerves [6]. These reports prompted device modifications and technique adjustments.

Due to safety concerns, the Food and Drug Administration mandated that healthcare providers undergo training and certification before using the Nexplanon® in practice [7, 8]. Current training recommends the Nexplanon® be placed subdermal and “at the inner side of the non-dominant upper arm about 8-10 cm (3-4 inches) above the medial epicondyle of the humerus, avoiding the sulcus (groove) between the biceps and triceps muscles” [9, 10]. Some providers use a different placement site, over the triceps muscle, to place the implant even further away from neurovascular structures of the medial arm, located between the triceps and biceps [11]. To help guard against deep placement, the proper technique involves insertion at an angle of less than 30° [10]. Furthermore, the newly added plastic barrier over the insertion needle is designed to guide the Nexplanon® into the superficial layer below the dermis [12, 13].

Proper removal technique is also important in avoiding complications. The safest method of extraction involves definitive palpation of the device immediately before attempting in-office removal [10]. Devices may be non-palpable because they were placed deeply or because they have migrated. Contraceptive devices placed in the arm have migrated as far as the shoulder, axilla, chest wall, and even the pulmonary arteries [9]. From the hand surgeon’s perspective, implant migration is a well-documented complication and is known to occur even when an implant is originally placed into solid bone [14]. Merck acknowledges that Nexplanon® migration is a risk and recommends that if the device is not palpable at the time of planned removal, X-ray, computed tomography (CT), ultrasound, or even magnetic resonance imaging (MRI) can be used to aid location [10].

Even with the mandated training, technique guidelines, and design improvements, serious complications can still arise. Merck maintains a database of deep implants and complex removals [15]. The complication we present is the first case reported of ulnar nerve injury during attempted in-office removal of a deep Nexplanon®. The patient required formal surgical exploration for implant removal, resection of an ulnar nerve traumatic neuroma, and reconstruction of the ulnar nerve.

## Case report

A 21-year-old woman presented to our hand and peripheral nerve clinic 4 months after attempted Nexplanon® removal from her left arm. The patient reported that neither she, nor her nurse practitioner (NP), was confidently able to feel the Nexplanon® before the attempted removal. Imaging studies to confirm location of the Nexplanon® were not performed. The patient remembered a small incision being made at the site of insertion after local anesthetic was injected. The provider was not immediately able to find the Nexplanon®, but after exploring the local area, did grasp another structure in her arm. The patient felt an “electric shock” sensation that radiated down to her medial elbow as the NP pulled. No further attempts to remove the Nexplanon® were undertaken.

Immediately after the removal attempt, the patient had complete numbness in her small and ring fingers. She returned for follow up to her NP. As months progressed, the numbness did not improve, and she began to notice wasting of her hand muscles and weakness in her grip. At almost 4 months after the attempted removal, her NP ordered a nerve conduction study which showed 50% loss of ulnar nerve function.

**Fig. 1:**
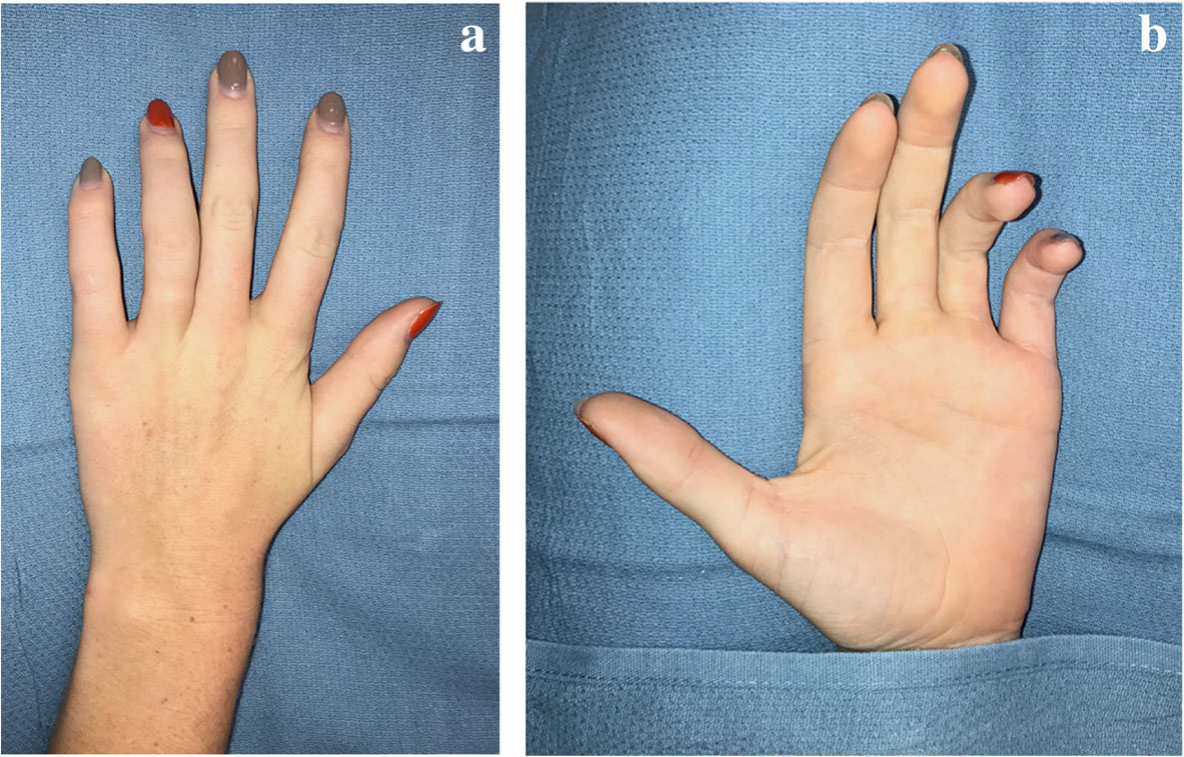
Clinical appearance on initial presentation to the hand surgery service The patient had wasting of the ulnar innervated intrinsic muscles of the hand between the metacarpals. **a** The patient also had an ulnar claw hand deformity. When the ulnar innervated intrinsic muscles cannot fire, there is extension at the metacarpalphalangeal (MCP) joints and flexion at both the proximal and distal interphalangeal (IP) joints in the ring and small fingers (**b**)

**Fig. 2:**
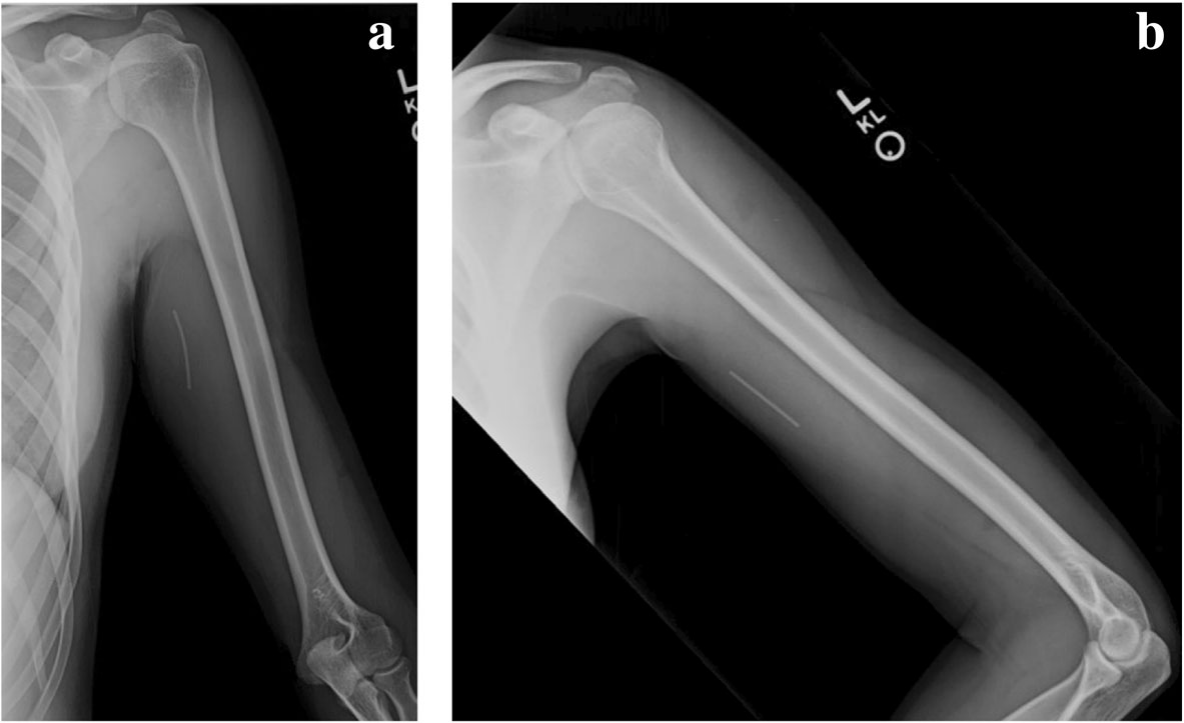
AP (**a**) and lateral (**b**) radiograph views of the left humerus show the radio-opaque implant located at the level of the proximal to mid humeral shaft, 16.5 cm proximal to the medial epicondyle

On presentation to the office, she had classic signs of severe, chronic ulnar nerve injury: wasting of the ulnar nerve-innervated intrinsic muscles of the hand, a claw position of the ring and small fingers, and dense numbness in an ulnar sensory nerve distribution (Fig. 1). On examination of her arm, there was a well-healed incision with surrounding scar tissue from the extraction attempt. The Nexplanon® was not palpable. X-rays showed the radio-opaque Nexplanon® at the junction of the proximal and middle thirds of the humeral shaft with the most distal end 16.5 cm proximal to the medial epicondyle (Fig. 2).

**Fig. 3:**
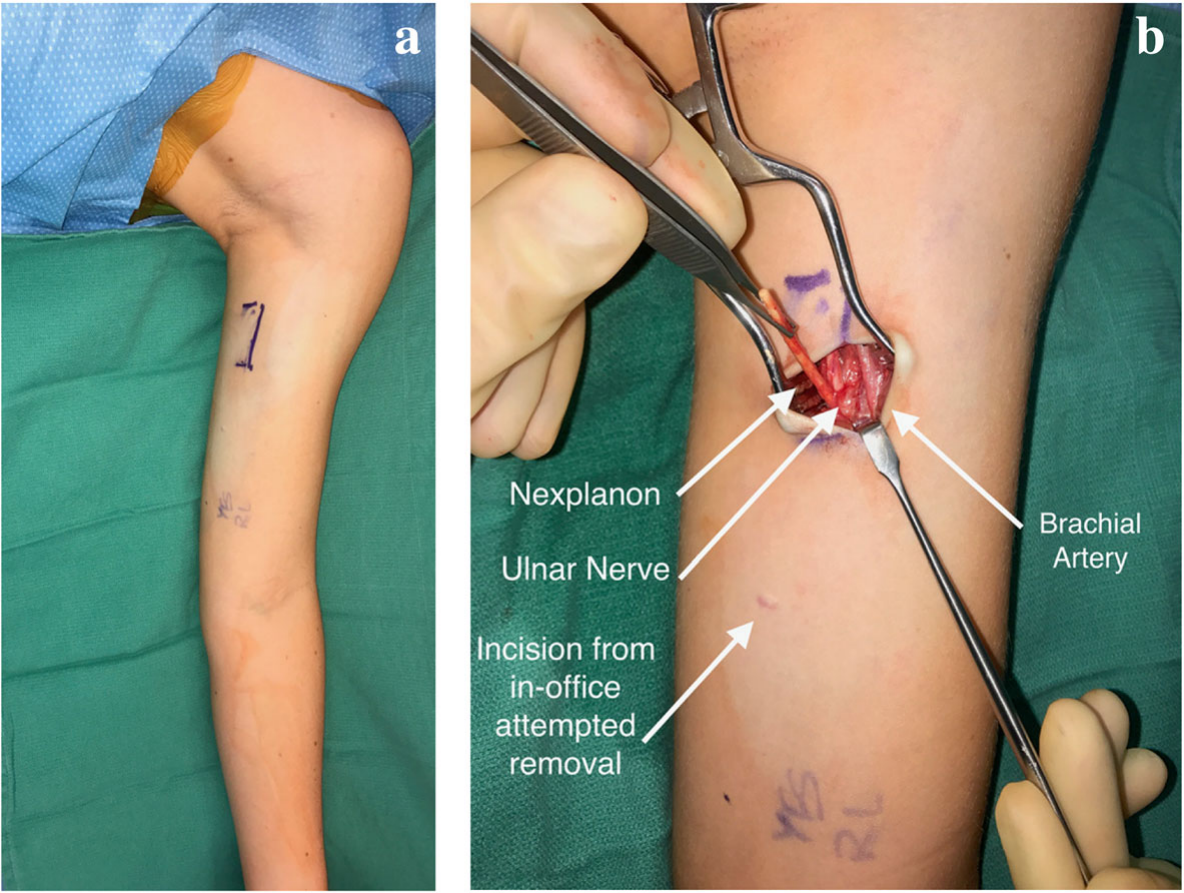
Nexplanon removal. The location of the Nexplanon was marked using intra-operative fluoroscopy before incision (**a**). Surgical removal of the Nexplanon was then undertaken at this location (**b**). The ulnar nerve was just deep to the Nexplanon and the brachial artery was in close proximity. Note the location of the Nexplanon in relation to the incision used for the attempted in-office removal

Given the patient’s history, physical exam, and nerve tests, timely surgical intervention was recommended. In the OR, the upper extremity surgery team used fluoroscopy to mark the location of the Nexplanon® (Fig. 3). On surgical exploration, the Nexplanon® was found deep to the brachial fascia of the arm and in direct contact with the ulnar nerve. Less than five millimeters away was the undamaged brachial artery--the main blood supply to the arm, forearm, and hand. The Nexplanon® was removed using microsurgical instruments.

**Fig. 4:**
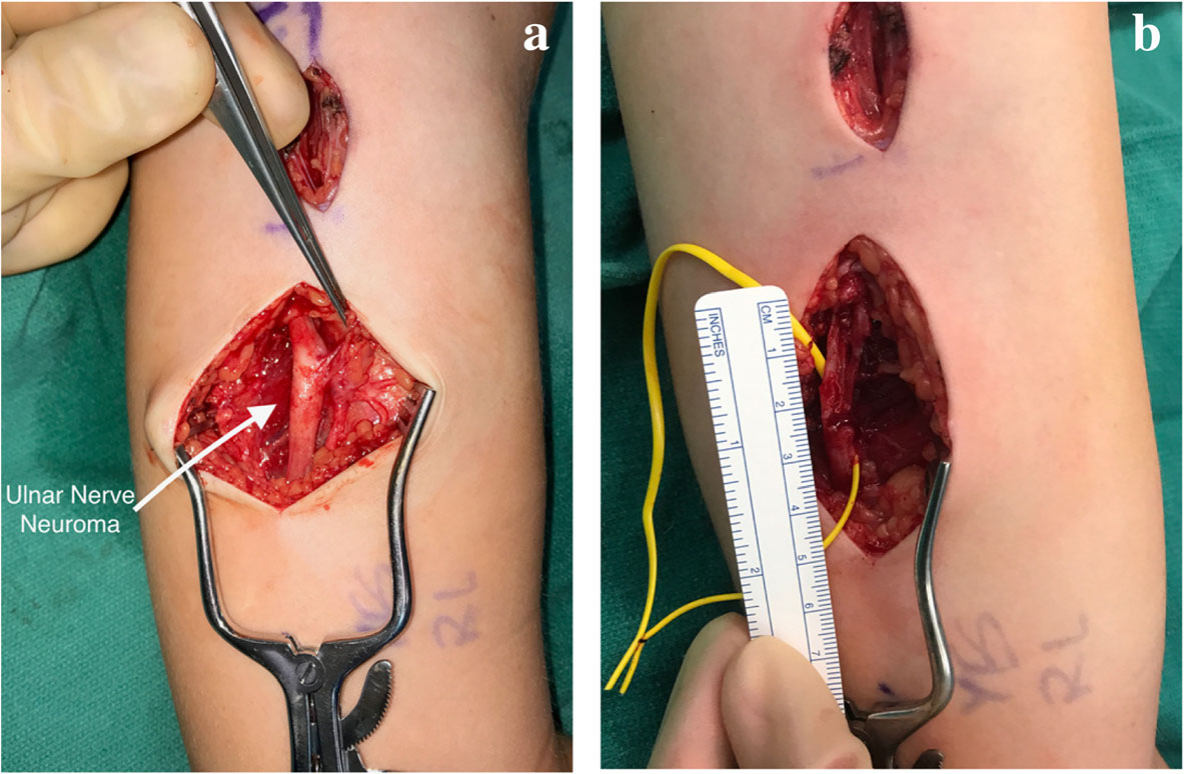
Identification and resection of ulnar nerve neuroma. An ulnar nerve neuroma in continuity was identified by fusiform swelling and fibrotic nerve (**a**). After resection of the traumatic neuroma, 3 undamaged deep ulnar nerve fascicles were left intact, but a 3 cm gap was left in the majority of the nerve (**b**)

Because of the patient’s dense ulnar nerve palsy, the ulnar nerve at the level of the attempted extraction was also explored. Dissection showed that the ulnar nerve had been severely damaged at this level (Fig. 4). Nerve injury can take many forms; this patient’s injury was a neuroma-in-continuity whereby the ulnar nerve was still one solid, longitudinal structure, but contained an abnormal portion, filled with scar tissue and damaged nerve fascicles that could not conduct electrical signal. The surgical team confirmed the neuroma’s inability to conduct via intra-operative electrical stimulation. The appearance of her neuroma-in-continuity was classic: the neuroma was fusiform in shape and felt thickened and hard, unlike the proximal and distal, soft and pliable uninjured nerve (Fig. 4a).

**Fig. 5:**
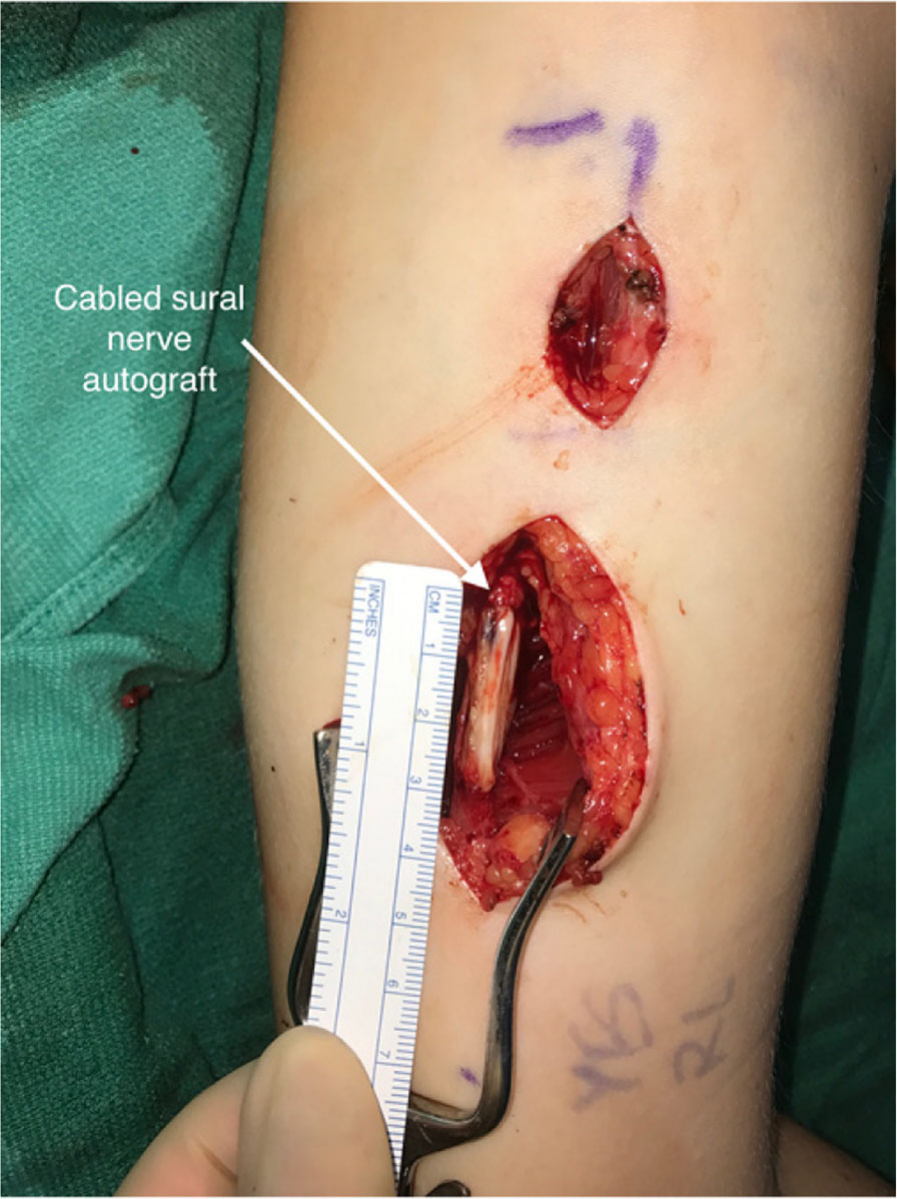
Ulnar nerve reconstruction using cabled autologous sural nerve graft. Sural nerve autograft was harvested from the patient’s leg and used to create a reversed, cabled nerve graft of matching length and diameter. It was placed into the ulnar nerve defect using a surgical microscope, 9–0 Nylon sutures, and fibrin glue

The upper extremity surgeons treated the neuroma-in- continuity with microsurgical resection, followed by reconstruction. After the damaged, scarred nerve was removed, there was a 3 cm gap between healthy sections of the ulnar nerve (Fig. 4b). The patient retained three uninjured nerve fascicles which made up less than 20% of the normal diameter of the nerve. The healthy fascicles were dissected free and preserved (Fig. 5b). To bridge the nerve gap, the patient’s sural nerve was harvested from her lower leg, cut into 3 cm long segments and bundled together to recreate the caliber and fascicles of the resected ulnar nerve (Fig. 5). This cabled sural nerve autograft was sutured into place using a surgical microscope and 9–0 Nylon sutures.

At 7 months after nerve reconstruction, the patient had weak grade 2/5 function of ulnar innervated muscles. She still had dense numbness in an ulnar nerve distribution. She did have an advancing Tinel’s sign on physical exam and reported intermittent paresthesias in an ulnar nerve distribution in her hand.

## Discussion and conclusions

The presented case is an example of an uncommon, but serious complication from implantable contraception. Peripheral nerve injury from any cause often carries a poor prognosis and causes significant disability for patients. For this patient, both deep implantation and in-office removal of a non-palpable device contributed to the iatrogenic nerve injury. The medial arm is a dense anatomic area where precise device implantation and extraction are required to maximize patient safety. Our patient’s limited recover 7 months after nerve reconstruction is unfortunately not uncommon: the prognosis for recovery after repair or reconstruction is particularly poor for the ulnar nerve [16, 17]. Prompt diagnosis and treatment is crucial to preserve treatment options and maximize a patient’s outcome. It is also important to keep in mind that delayed or incomplete treatment of nerve injuries, particularly of iatrogenic nerve injuries, can result in significant liability [18, 19].

Peripheral nerves of the arm can be at risk with either insertion or extraction of a contraceptive implant into the arm. The majority of cases have been described with older implantable devices. Specifically, Norplant has been reported to cause transient paresthesias that resolve with removal [20, 21]. Norplant removal has be associated with ulnar nerve injury and with significant scar tissue around the ulnar nerve causing nerve compression with motor and sensory symptoms [22]. Implanon® (Organon International INC, Roseland, NJ) insertion and removal has been linked to sensory-only medial antebrachial cutaneous nerve injury, medial cutaneous nerve of the forearm injury, and transient ulnar nerve sensory deficit after device removal [6, 8, 23]. And, as in our case, the currently available Nexplanon® with it’s updated delivery system and specific insertion and removal technique has been associated with median nerve injury in two cases [3]. Other recently reported cases do not mention the specific device used, but describe injury to the median nerve, medial antebrachial cutaneous, and ulnar nerves in a single patient and structural injury to the ulnar nerve in another patient [4, 5].

We emphatically agree with the current manufacturer recommendations: insertion should avoid the sulcus between the biceps and triceps where the median nerve, ulnar nerve, brachial artery and vein are located [10]. Furthermore, our case strongly supports the manufacturer’s recommendation that extraction should not be attempted without knowing the exact location of the device [10]. It is the senior author’s opinion that in-office placement and removal should be performed or directly supervised by an experienced physician who has undergone the appropriate implant-based training.

Since deep implantation and migration are possible, imaging studies should be used to precisely localize migrated or non-palpable devices before removal. In one paper describing operative removal of 28 implantable contraceptive devices, 30% of implants had migrated from the insertion site with 37% lying intramuscular and 11% lying in the neurovascular sheath [8]. Imaging studies should also be done for any patient who develops neurologic symptoms. X-ray, CT, MRI, and ultrasound can all be used to precisely identify an implant’s location. X-ray and ultrasound are both inexpensive, accessible, and non-invasive imaging modalities. While ultrasound offers a zero-radiation technique for localization, X-ray machines and radiologist interpretation are widely available and relatively inexpensive. Although X-ray does involve ionizing radiation, two standard views of the humerus only exposes a patient to 0.001 mSv of radiation [24]. This is the same amount of radiation an average US habitant sees by simply being exposed to our environment for 3 h [24].

Multidisciplinary care involving family planning practitioners and peripheral nerve surgeons for complex removal improves patient care and optimizes safety [25]. Nerve surgeons can have a hand surgery, orthopaedic surgery, plastic surgery, or neurosurgery background. Symptoms that can indicate an iatrogenic nerve injury at the time of placement or removal include electric or shock-like pain, numbness, or weakness in the distribution of a peripheral nerve. Physical exam findings can include decreased sensation to touch and hand or forearm weakness. Late signs of untreated nerve injury include visible muscle wasting or abnormal posturing such as our patient’s ulnar claw hand.

Prompt referral to a peripheral nerve surgeon is crucial because the motor end plates and end target muscles irreversibly degenerate without nerve input [26]. After nerve repair or grafting, nerves regenerate at a rate of approximately 1 mm per day [26]. As a rule of thumb, if the newly advancing axons do not reach the muscle by 12 months after injury, the damage is permanent and no meaningful functional recovery is made [26].

Even though there are surgical options for timely nerve repair and reconstruction, normal sensation and strength almost never return. Loss of ulnar nerve function is devastating for patients as the ulnar nerve is responsible for extrinsic and intrinsic hand muscles, as well as crucial hand sensation [27]. Sural nerve autograft is the current gold standard in major peripheral nerve reconstruction. Taking the sensory sural nerve from the leg universally results in lateral foot numbness [26]. In addition, a small percentage of patients have lasting neuropathic pain from this iatrogenic sural nerve injury [26].

In conclusion, Nexplanon® related major peripheral nerve injuries are an uncommon but possible complication. We recommend documenting the precise location of the implant with careful physical exam after insertion and before extraction. If a patient develops nerve symptoms in the setting of a non-palpable implant or migrated device, imaging and prompt referral to a peripheral nerve and upper extremity surgeon is strongly recommended. Experienced referral centers can be located by contacting the manufacturer.
